# Adjunctive Probio-X Treatment Enhances the Therapeutic Effect of a Conventional Drug in Managing Type 2 Diabetes Mellitus by Promoting Short-Chain Fatty Acid-Producing Bacteria and Bile Acid Pathways

**DOI:** 10.1128/msystems.01300-22

**Published:** 2023-01-23

**Authors:** Ye Chen, Xin Shen, Teng Ma, Xia Yu, Lai-Yu Kwok, Yalin Li, Zhihong Sun, Dongmei Li, Heping Zhang

**Affiliations:** a Inner Mongolia People’s Hospital, Hohhot, Inner Mongolia, China; b Inner Mongolia Key Laboratory of Dairy Biotechnology and Engineering, Inner Mongolia Agricultural University, Hohhot, Inner Mongolia, China; c Key Laboratory of Dairy Products Processing, Ministry of Agriculture and Rural Affairs, Inner Mongolia Agricultural University, Hohhot, Inner Mongolia, China; d Key Laboratory of Dairy Biotechnology and Engineering, Ministry of Education, Inner Mongolia Agricultural University, Hohhot, Inner Mongolia, China; University of California—San Diego

**Keywords:** type 2 diabetes mellitus, probiotics, metformin, gut microbiome, short-chain fatty acids, bile acids

## Abstract

Metformin is a common drug for the management of type 2 diabetes mellitus; however, it causes various adverse gastrointestinal effects, especially after prolonged treatment. It is thus of interest to identify an adjuvant treatment that synergizes with the efficacy of metformin while mitigating its adverse effects. Since previous evidence supports that the gut microbiota is a target of metformin, this study investigated the beneficial effect and mechanism of the coadministration of probiotics with metformin in the management of type 2 diabetes mellitus by conducting a 3-month randomized, double-blind, placebo-controlled clinical trial (*n* = 27 and 21 in the probiotic and placebo groups, respectively, who completed the trial). Clinical results showed that the coadministration of probiotics with metformin significantly reduced glycated hemoglobin compared with metformin taken alone (*P < *0.05). Metagenomic and metabolomic analyses showed that the coadministration of probiotics increased the abundance of gut short-chain fatty acid (SCFA)-producing bacteria and bile acids. Significantly or marginally more bile acids and related metabolites were detected in the probiotic group than in the placebo group postintervention. Taken together, the results of our study showed that the coadministration of probiotics with metformin synergized with the hypoglycemic effect in patients with type 2 diabetes mellitus, which was likely through modulating the gut microbiome and, subsequently, SCFA and bile acid metabolism. Our findings support that cotreatment with probiotics and metformin is beneficial to patients with type 2 diabetes mellitus.

**IMPORTANCE** Metformin causes variable adverse gastrointestinal effects, especially after prolonged treatment. We found that cotreatment with Probio-X and metformin for the management of type 2 diabetes mellitus may promote gut SCFA-producing bacteria and the levels of specific bile acids, thus increasing the secretion of related gastrointestinal hormones and ultimately improving glucose homeostasis.

## INTRODUCTION

Type 2 diabetes mellitus (T2DM) is a chronic metabolic endocrine disease characterized by elevated blood glucose levels. The typical symptoms of diabetes are “three more and one less,” namely, polyuria, polydipsia, polyphagia, and weight loss. Modern lifestyle changes in the last decades have greatly increased the number of patients (1). At present, there are over 500 million people with diabetes worldwide. In the past 10 years, the incidence of T2DM in the United States has increased by 7.1% annually, from 9 to 12.5 per 100,000 population during the last 10 years (2). The prevalence of diabetes in China rose from 0.7% in 1980 to 12.8% in 2017, with more than one-third of adults having prediabetes (3). Diabetes is usually accompanied by a series of complications that seriously endanger human health, such as diabetic nephropathy and cardiovascular and cerebrovascular diseases (4, 5).

Currently, biguanides, α-glucosidase inhibitors, and incretin-based drugs are commonly used in diabetes treatment (6). Metformin is the drug most frequently used for treating T2DM, but its specific hypoglycemic mechanism is still unclear. Generally, it is believed that metformin exerts its antihyperglycemic action mainly by inhibiting mitochondrial respiratory complex I, increasing the level of AMP, and activating AMP-activated protein kinase to reduce hepatic glucose production (7). However, more and more studies have shown that intestinal microbes may play an important role in the hypoglycemic mechanism of metformin. For example, previous studies have shown that the alpha diversity of the gut microbiota was generally lower in patients with T2DM than in healthy individuals (8, 9), and the relative abundances of *Firmicutes* and *Actinobacteria* increased, while those of *Bacteroidetes* decreased (10). Metformin lowers the blood sugar of T2DM patients, accompanied by significant changes in gut microbes, including a significant decrease in the abundance of Bacteroides fragilis (11), suggesting a role of the gut microbiota in reducing blood sugar. Existing evidence supports that the gut microbiota regulates metabolic diseases by regulating bile acids (BAs). Bile acids and their metabolites are important biologically active ligands that interact with intestinal receptors to regulate metabolism (12). In BA metabolism, the liver converts cholesterol into primary BAs, which are absorbed by the apical sodium-dependent BA transporter into enterocytes (13). After entering the gut, BAs are chemically modified by gut microbes, and bacterium-modified BAs can act as signaling molecules to further interact with gut microbes (14). A recent combined *in vivo* and *in vitro* study demonstrated that metformin could improve metabolic disorders such as hyperglycemia via reducing the gut level of Bacteroides fragilis, which then increased specific BAs, inhibited intestinal farnesoid X receptor signals, and increased blood glucagon-like peptide 1 (GLP-1), and these actions together could improve blood sugar homeostasis (11). These reports consistently support that metformin targets the BA-modulating gut microbiota to affect the clinical manifestations of T2DM.

In clinical situations, patients with diabetes are treated with a single or a combined regimen depending on the severity of hyperglycemia and the target of symptom management. However, diabetes medications inevitably cause some side effects or discomfort, especially when a combined regimen is administered. For example, metformin may cause nausea, gas, bloating, diarrhea, vitamin B_12_ deficiency, and an upset stomach, and liraglutide may cause nausea, diarrhea, vomiting, decreased appetite, indigestion, and constipation. Metformin is usually the first drug prescribed for the management of T2DM, and coprescription with other drugs like liraglutide offers added treatment effects. A previous randomized double-blind trial conducted on adolescents with T2DM reported significantly greater efficacy in glycemic control over 52 weeks, evidenced by the more obvious reduction in the mean glycated hemoglobin and fasting plasma glucose (FPG) levels, by the combined application of metformin and liraglutide than in the placebo group receiving only metformin but not liraglutide. Both groups of patients complained about treatment-associated side effects; however, the added beneficial effects of liraglutide application were at the cost of a higher frequency of gastrointestinal adverse events (15). Therefore, it is very important to take into consideration the adverse effects when combined regimens are prescribed, and it would be very meaningful to explore treatments that not only enhance the clinical efficacy of metformin but also are safe to take, with minimal adverse effects.

Probiotics are defined as living microorganisms that, when given in sufficient amounts, confer health benefits to the host (16). Probiotic administration has been shown to improve clinical indicators of T2DM in a number of animal and human randomized controlled trials (RCTs). Ke et al. showed that intervention with synbiotics (Bifidobacterium animalis subsp. *lactis*, Lactobacillus paracasei subsp. *paracasei* DSM 46331, and oat β-glucan) could significantly reduce weight, blood glucose, lipids, and other clinical indicators in diet-induced obese mice (17). Supplementation with multistrain probiotics was found to reduce the levels of fasting blood glucose, the levels of glycosylated hemoglobin, and the insulin resistance index in patients with T2DM (18). Probiotics or synbiotics were also found to significantly reduce the level of glycosylated hemoglobin in patients with prediabetes (19). It is worth mentioning that a meta-analysis of nine RCTs found that compared with traditional yogurt, probiotic yogurt did not significantly improve fasting blood glucose, fasting insulin, insulin resistance, and other indicators of T2DM/obesity in patients (20). Although many studies have shown that probiotics and related products offer beneficial effects in mitigating T2DM, consistent guidelines of how such treatments could be used in clinical situations have not been clearly laid out, in particular the strains to be used, probiotic dosage, and intervention time, due to variations in the clinical effects seen between studies and across individuals. Such variations could be the result of multiple factors, including the strain specificity effect of probiotics or physiological differences between individuals. Therefore, large-scale and high-quality clinical trials are still needed to explore potential hypoglycemic candidate strains.

The probiotic product Probio-X comprises five different probiotic strains, Lactobacillus casei Zhang, Lactobacillus plantarum P-8, Lactobacillus rhamnosus Probio-M9, Bifidobacterium animalis subsp. *lactis* M8 (Probio-M8), and Bifidobacterium animalis subsp. *lactis* V9. Previous clinical studies have shown various beneficial effects of these strains. For example, Lactobacillus casei Zhang can reduce fat accumulation and central obesity (21). Bifidobacterium animalis subsp. *lactis* V9 can interact with intestinal hormones and affect the secretion of sex hormones in the pituitary gland-hypothalamus through the intestine-brain axis, thus relieving polycystic ovary syndrome (22). Lactobacillus plantarum P-8 can reduce the levels of inflammatory factors and improve cognition (23). Lactobacillus rhamnosus Probio-M9 has a good curative effect on tumor prevention and treatment (24). Probio-M8 can reduce the level of trimethylamine oxide and improve related symptoms of coronary heart disease (25). Probio-X contains these five bacteria and has been used in a number of clinical trials. Previous studies have shown that Probio-X maintains intestinal microbiota homeostasis (16) and relieves hyperlipidemia by regulating lipid metabolism (Huan Wang, Cuicui Ma, Yan Li, Lei Zhang, Alima, Chengcong Yang, Feiyan Zhao, Haifeng Han, Dongyang Shang, Fan Yang, Yuying Zhang, Heping Zhang, Zhihong Sun, Ruifang Guo). Our previous study found that Probio-M8 could mitigate T2DM symptoms in a hyperglycemic mouse model (Ye Chen, Yaxin Zhao, Xin Shen, Feiyan Zhao, Jinxin Qi, Zhi Zhong, Dongmei LI). Therefore, the compound probiotic product Probio-X was selected for the current trial.

Probiotics are generally not considered medications; however, provided the previously demonstrated T2DM symptom alleviation effects, we designed the current 3-month RCT to investigate the synergistic effect of combined treatment with metformin and probiotics in patients with T2DM (*n* = 58 total T2DM patients recruited initially; *n* = 29 in each of the probiotic and placebo groups [random group allocation]). The primary outcomes were multiple clinical indicators of T2DM, including fasting blood glucose, glycosylated hemoglobin, glucose tolerance, and islet function. In addition, changes in the fecal metagenomes and metabolomes of the patients were monitored before and after the intervention. The second purpose of this study was to perform bioinformatics analyses on the changes in the patients’ gut microbiota, clinical indicators, and metabolites to reveal the potential hypoglycemic mechanism of the combined regimen in improving T2DM. Uncovering such a hypoglycemic mechanism would be an important step in the development of clinical guidelines for the use of probiotics as an adjunctive treatment with conventional medications for the management of T2DM.

## RESULTS

### Demographic data.

**FIG 1:**
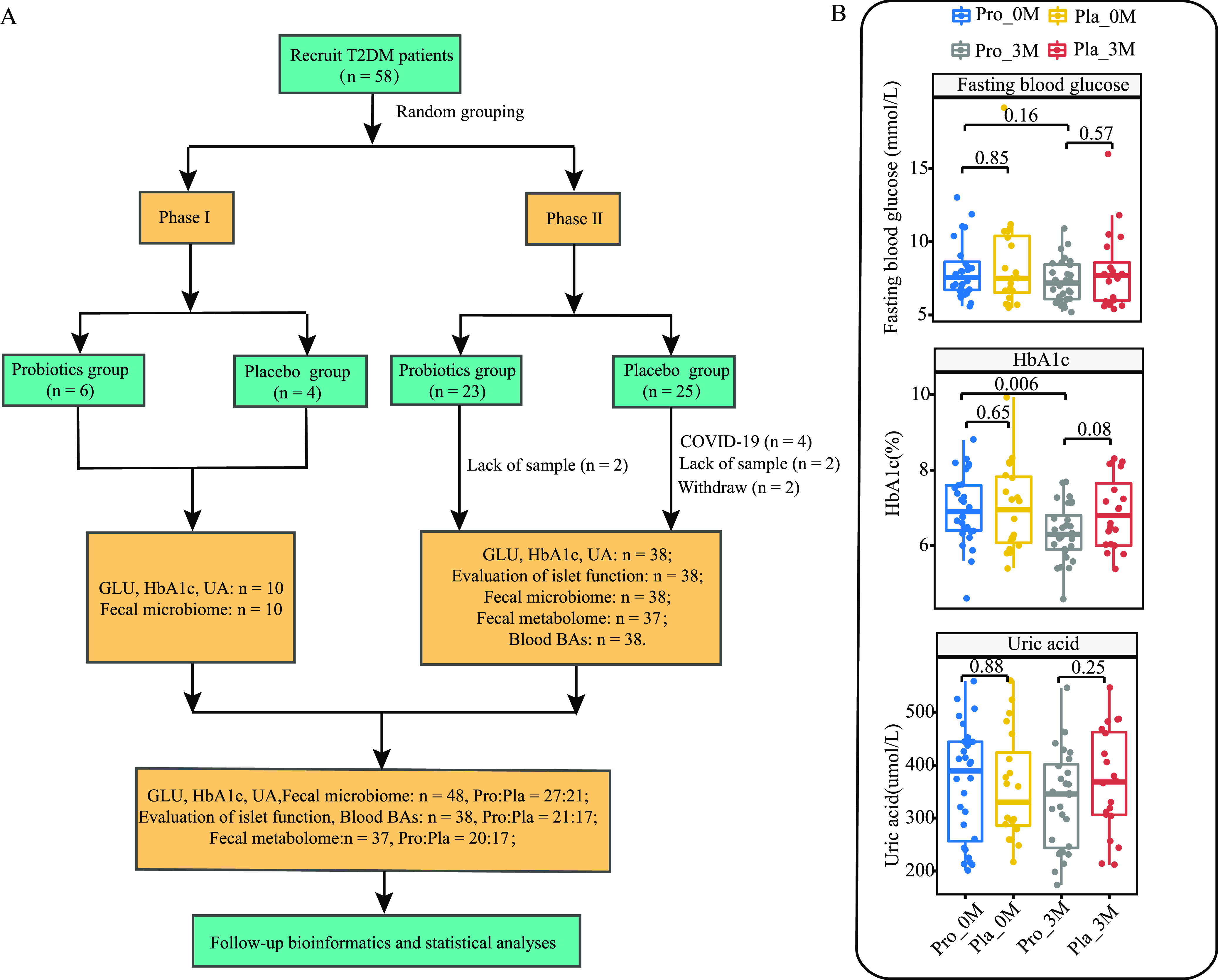
Trial design and clinical indicators of T2DM. (A) Flow diagram of the clinical trial. GLU, glucose. (B) Changes in the levels of fasting plasma glucose, hemoglobin A1c (HbA1c), and blood uric acid (UA) in the probiotic (Pro) and placebo (Pla) groups before (0M) and after (3M) the 3-month intervention. A Wilcoxon test and a *t* test were used to evaluate horizontal differences between the probiotic and placebo groups at the same time point and longitudinal differences between months 0 and 3 for the same group. *P* values generated by pairwise comparisons are given.

A total of 58 patients with T2DM were enrolled, including patients in the first phase (*n* = 10) and the second phase (*n* = 48), and only 48 patients completed the clinical trial (probiotic group, *n* = 27; placebo group, *n* = 21) (Fig. 1A). Subjects were aged 48.70 ± 11.11 years (probiotic group) and 46.90 ± 11.25 years (placebo group). There was no significant difference in baseline age, body weight, and waist circumference between the two groups (*P *= 0.60, *P *= 0.44, and *P *= 0.16, respectively) (see Table S1 in the supplemental material). All patients received metformin (metformin hydrochloride extended-release tablets; Bristol-Myers Squibb Company) (0.75 to 1.5 g/day in 3 doses) (Table S1) with 2 g of placebo material or probiotics accordingly. In the first phase, only fasting blood glucose, glycosylated hemoglobin, uric acid, and fecal metagenome samples were collected. Therefore, the fecal metabolism of patients in the first phase was not analyzed. In the second phase, 10 individuals (2 and 8 individuals from the probiotic group and the placebo group, respectively) failed to be followed up, and one individual’s sample was insufficient. Therefore, only samples from 37 individuals were analyzed for fecal metabolism in this study (probiotic group, *n* = 20; placebo group, *n* = 17) (Fig. 1A).

10.1128/msystems.01300-22.2 TABLE S1 Information on the subjects. Download Table S1, PDF file, 0.1 MB. Copyright © 2023 Chen et al. 2023 Chen et al. https://creativecommons.org/licenses/by/4.0/ This content is distributed under the terms of the Creative Commons Attribution 4.0 International license.

### Coadministration of probiotics enhanced the decreases in blood sugar and glycosylated hemoglobin levels but not uric acid and serum lipid levels.

Fasting blood glucose, glycosylated hemoglobin, and uric acid are important indicators for diagnosing diabetes. Our results showed that the coadministration of Probio-X with metformin for 3 months significantly reduced the glycosylated hemoglobin level (*P < *0.05), while no significant change was observed in the placebo group (Fig. 1B). It is worth noting that the fasting blood glucose levels of probiotic recipients showed a downward trend. A high level of uric acid usually increases the risk of the development of kidney disease in diabetes patients. Although there was no significant difference between groups or time points, the uric acid level of the probiotic group showed a nonsignificant decrease after the 3-month intervention (Fig. 1B), while the placebo group showed the opposite trend. We monitored four blood lipid indicators, triglyceride (TG), high-density lipoprotein cholesterol (HDL-C), low-density lipoprotein cholesterol (LDL-C), and total cholesterol (CHOL), in all patients, and no significant differences were found in these blood lipid indices between the two groups (Table S2). These results suggested that the coadministration of Probio-X and metformin significantly enhanced the hypoglycemic effect compared to metformin taken alone, and the hypoglycemic effect was independent of the monitored serum lipid indicators.

10.1128/msystems.01300-22.3 TABLE S2 Blood lipid indices in diabetes patients before and after the probiotic/placebo intervention. Download Table S2, PDF file, 0.1 MB. Copyright © 2023 Chen et al. 2023 Chen et al. https://creativecommons.org/licenses/by/4.0/ This content is distributed under the terms of the Creative Commons Attribution 4.0 International license.

### Coadministration of probiotics enhanced insulin secretion and improved pancreatic islet function.

**FIG 2:**
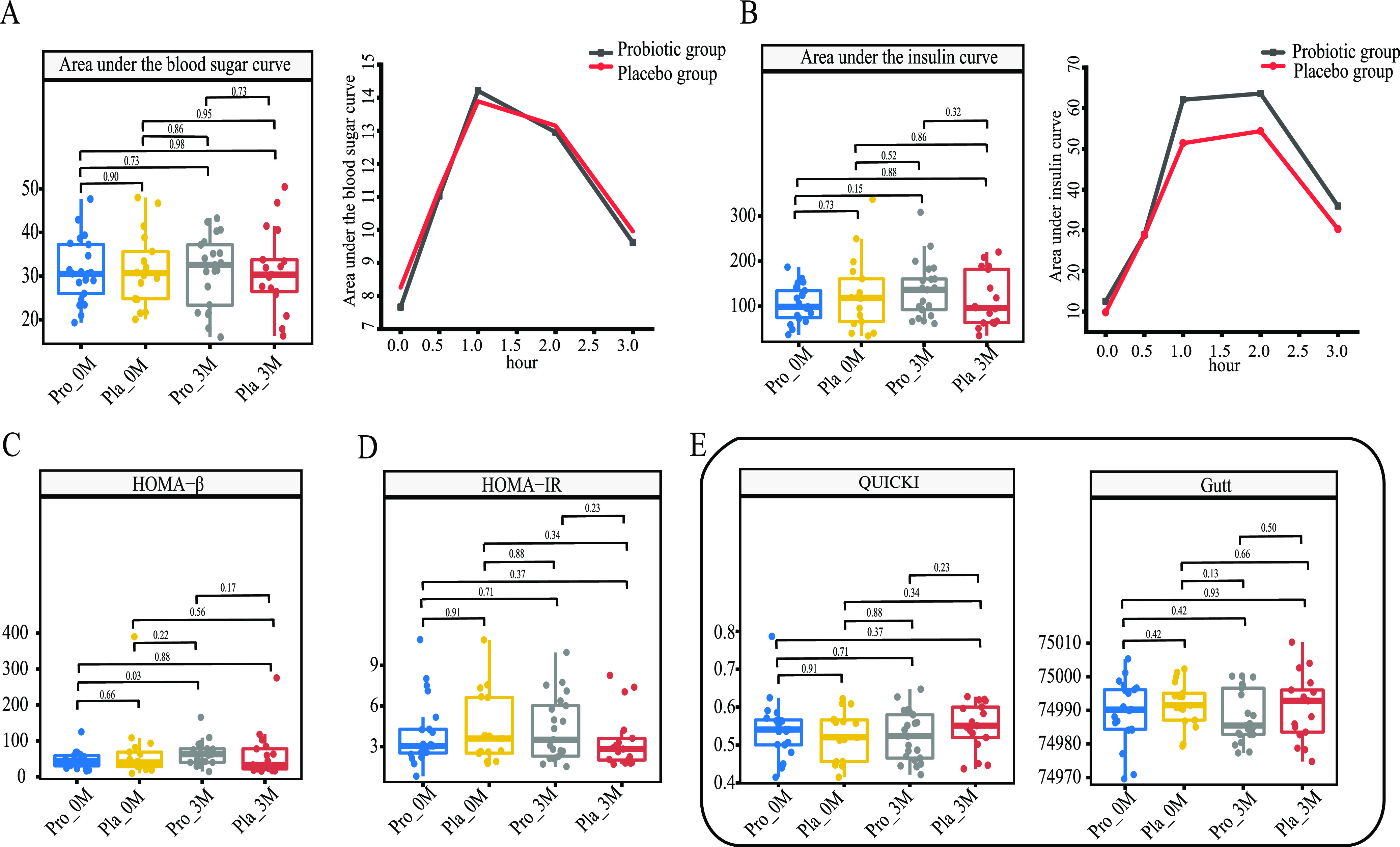
Glucose tolerance and islet function evaluation. (A and B) Differences in the areas under the curve of blood glucose (A) and insulin (B). (C) Homeostasis model assessment beta (HOMA-β). (D) Homeostasis model assessment-estimated insulin resistance (HOMA-IR). (E) Quantitative insulin sensitivity check index (QUICKI) and Gutt index (insulin sensitivity index [ISI_0,120_]) between the probiotic (Pro) and placebo (Pla) groups before (0M) and after (3M) the 3-month intervention. *P* values were generated by pairwise comparisons (Wilcoxon test).

The oral glucose tolerance test (OGTT) is a glucose stress test that reveals the function of pancreatic islets and the body’s ability to regulate blood sugar (26). No significant differences were found in the areas under the blood glucose and insulin curves between groups or time points (Fig. 2A and B). The β-cell function index (represented by the homeostasis model assessment beta [HOMA-β] index) in the probiotic group increased significantly after the 3-month intervention (*P *= 0.03) (Fig. 2C), but there were no significant differences in the insulin resistance and insulin sensitivity indices (Fig. 2D and E; Table S3). Our results indicated that Probio-X could increase insulin secretion and improve islet function.

10.1128/msystems.01300-22.4 TABLE S3 Evaluation of islet function in diabetes patients before and after the probiotic/placebo intervention. Download Table S3, PDF file, 0.1 MB. Copyright © 2023 Chen et al. 2023 Chen et al. https://creativecommons.org/licenses/by/4.0/ This content is distributed under the terms of the Creative Commons Attribution 4.0 International license.

### Coadministration of probiotics modulated the fecal microbiome composition but not the overall microbiota diversity.

**FIG 3:**
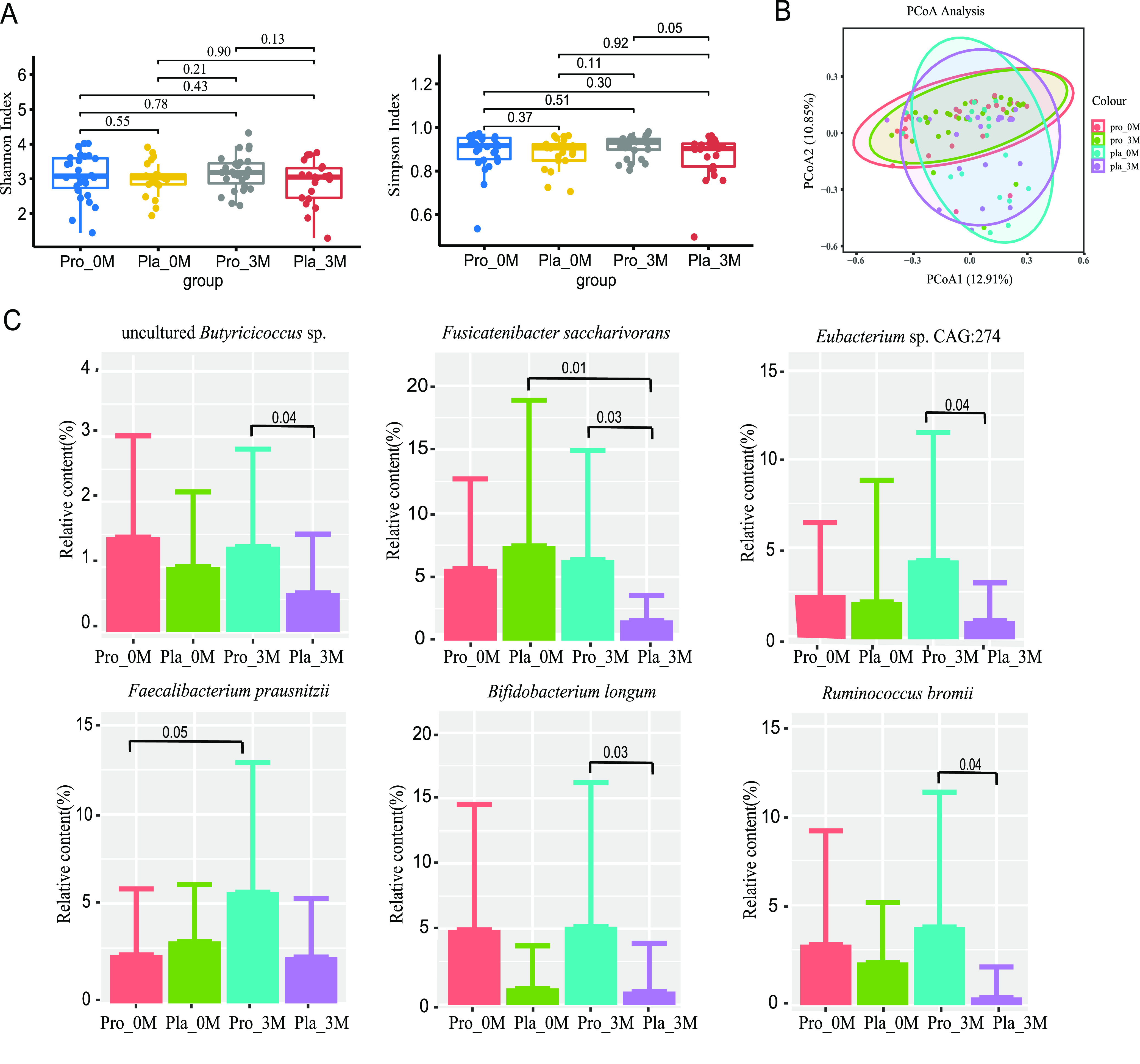
Microbial diversity and differentially abundant species-level genome bins (SGBs) between groups. (A) Shannon and Simpson diversity indices of the gut microbiota of the placebo (Pla) and probiotic (Pro) groups at month zero (0M) and after the 3-month intervention (3M). (B) Principal-component analysis (PCA) score plots for four subgroups. Symbols representing each subgroup are shown in different colors. (C) Significantly different SGBs between the probiotic and placebo groups at different time points. A *P* value of <0.05 was considered statistically significant.

This study analyzed the microbial metagenomes of 96 fecal samples (*n* = 48) (Table S4) at 0 and 3 months. In most cases, no significant differences were observed for alpha diversity (reflected by Shannon and Simpson diversity indices, except for Simpson diversity indices between the probiotic and placebo groups at month 3 [*P *= 0.05]) (Fig. 3A). Beta diversity was analyzed by principal-coordinate analysis (PCoA) (Fig. 3B). Symbols representing the four subgroups showed a high degree of overlap on the PCoA score plot, suggesting that there was a large commonality in the gut microbiota structure and composition between groups and time points, with no significant difference being detected by analysis of similarity (ANOSIM) (Table S5).

10.1128/msystems.01300-22.5 TABLE S4 Quality control information for the metagenomic data set. Download Table S4, PDF file, 0.2 MB. Copyright © 2023 Chen et al. 2023 Chen et al. https://creativecommons.org/licenses/by/4.0/ This content is distributed under the terms of the Creative Commons Attribution 4.0 International license.

10.1128/msystems.01300-22.6 TABLE S5 Analysis of similarity (ANOSIM) to evaluate differences in gut microbiota structures. Download Table S5, PDF file, 0.1 MB. Copyright © 2023 Chen et al. 2023 Chen et al. https://creativecommons.org/licenses/by/4.0/ This content is distributed under the terms of the Creative Commons Attribution 4.0 International license.

Next, postintervention changes in the gut microbiota composition at a finer taxonomic level were analyzed. Responsive species-level genome bins (SGBs) (SGBs that were not significantly different between the two groups at day 0 but became significantly different between time points or different treatments only after the intervention) were identified. One responsive SGB that was differentially abundant between month 3 and the baseline was identified in each group: Faecalibacterium prausnitzii in the probiotic group increased, and Fusicatenibacter saccharivorans in the placebo group decreased. At month 3, six differentially enriched SGBs were detected between the two groups: *Fusicatenibacter saccharivorans* and Ruminococcus bromii were significantly enriched in the placebo group compared with the probiotic group, and *Eubacterium* sp. strain CAG:274, Bifidobacterium longum, and an uncultured *Butyricicoccus* sp. were significantly enriched in the probiotic group compared with the placebo group (Fig. 3C; Table S6). Notably, some short-chain fatty acid (SCFA)-producing bacteria, such as Faecalibacterium prausnitzii and Bifidobacterium longum, were significantly enriched in the probiotic group (Fig. 3C).

10.1128/msystems.01300-22.7 TABLE S6 Significantly differential species-level genome bins (SGBs). Download Table S6, PDF file, 0.1 MB. Copyright © 2023 Chen et al. 2023 Chen et al. https://creativecommons.org/licenses/by/4.0/ This content is distributed under the terms of the Creative Commons Attribution 4.0 International license.

### Coadministration of probiotics modulated some fecal metabolites and serum BAs.

**FIG 4:**
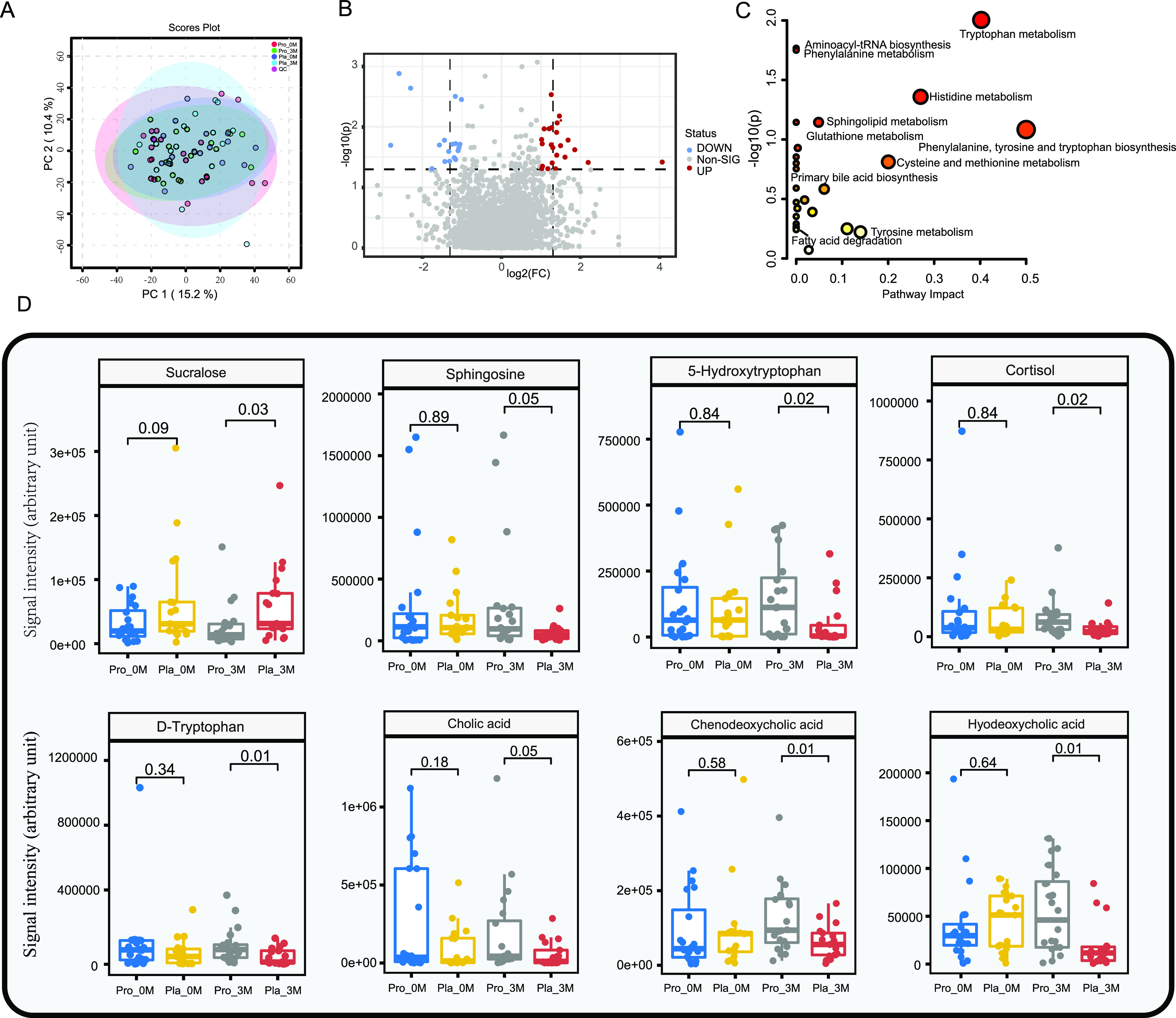
Changes in the fecal metabolome. (A) Principal-component analysis (PCA) score plot of the probiotic (Pro) and placebo (Pla) groups before (0M) and after (3M) the 3-month intervention. “QC” represents quality control samples. Samples from different subgroups are shown in different colors. (B) Volcano plot showing differential metabolites between the probiotic group and the placebo group at 3 months. Significantly increased metabolites (UP [upregulated]) (fold change [FC] of >2 and *P* value of <0.05) and significantly decreased metabolites (DOWN [downregulated]) (fold change of <0.5 and *P* value of <0.05) are represented. Non-SIG, nonsignificant. (C) Metabolic pathway enrichment analysis based on the differentially abundant metabolites. (D) Box plots showing relevant fecal metabolites responsive to probiotic intervention in diabetes patients. A total of 37 patients provided fecal samples at two consecutive time points for metabolomics analysis (probiotic group, *n* = 20; placebo group, *n* = 17). Statistical differences were assessed by a Wilcoxon test or a *t* test, and a *P* value of <0.05 was considered statistically significant.

Untargeted liquid chromatography-mass spectrometry (LC-MS)-based metabolomics analysis was performed to analyze targeted fecal metabolites. Data were analyzed by an unsupervised coordinate method, principal-component analysis (PCA); symbols representing the quality control (QC) samples were tightly clustered (Fig. 4A), indicating that the analytical instrument and conditions were stable and that the data generated were reliable. The differential metabolites were analyzed by a volcano plot (Fig. 4B), and 40 differential metabolites were found between the probiotic and placebo groups at month 3 (fold change >2.0 and *P* value <0.05 or fold change <0.5 and *P* value <0.05). All 40 differential metabolites were annotated to the corresponding compounds by searching the MS spectra across metabolite databases. The metabolic pathway analysis based on the 40 identified differential metabolites implied that these differential metabolic pathways between the probiotic and placebo groups belonged mostly to amino acid metabolic pathways (tryptophan metabolism, histidine metabolism, and phenylalanine, tyrosine, and tryptophan biosynthesis) and a few fatty acid metabolism pathways (e.g., sphingolipid metabolism) (Fig. 4C).

At month 3, the signal intensities of some metabolites were significantly (5-hydroxytryptophan, cortisol, d-tryptophan, chenodeoxycholic acid, and hyodeoxycholic acid [*P < *0.05 in all cases]) or marginally (sphingosine and cholic acid [*P *= 0.05 in both cases]) (Fig. 4D and Table S7) stronger in the probiotic group than in the placebo group, while sucralose showed the opposite trend (*P < *0.05) (Fig. 4D). Some of these substances (particularly cholic acid, hyodeoxycholic acid, and sphingosine) play important roles in primary bile acid biosynthesis and sphingolipid metabolism pathways. To further investigate the subjects’ metabolism of BAs, their serum BA levels were analyzed by LC-MS, and the serum BA composition exhibited only nonsignificant postintervention changes (Fig. S1).

10.1128/msystems.01300-22.1 FIG S1 Serum bile acid compositions of the probiotic (Pro) and placebo (Pla) groups before (0M) and after (3M) the 3-month intervention. Bile acid quantification was performed by liquid chromatography-mass spectrometry (LC-MS). Download FIG S1, PDF file, 0.2 MB. Copyright © 2023 Chen et al. 2023 Chen et al. https://creativecommons.org/licenses/by/4.0/ This content is distributed under the terms of the Creative Commons Attribution 4.0 International license.

10.1128/msystems.01300-22.8 TABLE S7 Differential fecal metabolites identified by liquid chromatography-mass spectrometry. Download Table S7, PDF file, 0.1 MB. Copyright © 2023 Chen et al. 2023 Chen et al. https://creativecommons.org/licenses/by/4.0/ This content is distributed under the terms of the Creative Commons Attribution 4.0 International license.

## DISCUSSION

Intestinal microbe dysbiosis is associated with the occurrence of various metabolic diseases such as T2DM, hypertension, and hyperlipidemia (27–29). Metformin is a common hypoglycemic drug for T2DM. The findings of multiple studies support that intestinal microbes might be one of the targets of metformin in patients (30). Probiotics have been shown to target the gut microbiota and mitigate gut dysbiosis, and probiotic consumption has been proposed as a new adjuvant treatment for T2DM. Therefore, this study investigated the added benefit of the administration of probiotics when taken with metformin in managing T2DM. Our results showed that the combined regimen significantly enhanced the hypoglycemic effect (significantly reduced the glycated hemoglobin level) compared with metformin taken alone. The coadministration of probiotics also nonsignificantly reduced the blood uric acid level in patients with T2DM. Our results suggested that the coadministration of probiotics synergized with the therapeutic effect of metformin in improving some of the T2DM-associated clinical parameters.

Insulin resistance is a pathological condition in which cells become nonresponsive to insulin, and the pancreas is signaled to secrete even more insulin to reduce blood sugar. Eventually, the cells become even more insulin resistant, causing elevated levels of both insulin and blood sugar. Our results suggested that the coadministration of probiotics was able to significantly improve the functional index of insulin β-cells, but no significant differences were found for the insulin resistance and insulin sensitivity levels (Gutt index and quantitative insulin sensitivity check index [QUICKI]) between the two groups. The homeostasis model assessment-estimated insulin resistance (HOMA-IR) index is the most common clinical indicator of insulin resistance, but its response to dynamic insulin secretion is limited (31); thus, we also used the Gutt index and QUICKI, yielding consistent results. On the other hand, a previous 6-month intervention study in patients with T2DM found that the administration of either Ecologic Barrier multistrain probiotics (multiple strains of *Bifidobacterium* and *Lactobacillus*) or placebo significantly reduced the HOMA-IR index (18). It is interesting to see the contrasting results between studies, which might be due to various factors such as the subjects’ personal factors, probiotic specificities, and dosage issues. Nevertheless, the current results showed that the coadministration of Probio-X with metformin improved some T2DM-associated indicators, although no significant change was observed in insulin resistance between the probiotic and placebo groups.

Some previous studies found that the hypoglycemic effect of metformin is obvious through oral intake but not via intravenous injection, implying that the mechanism of action of metformin is directly related to the gastrointestinal tract (32, 33). In this study, one hypothesis made was that the gut microbiome was one of the major targets of the blood sugar-lowering action of metformin. Such a hypothesis was based on evidence provided by previous studies reporting the possible role of metformin in regulating the structure of the gut microbiota and its metabolites, thereby affecting the physiological function of the host (34). Elbere et al. even proposed that the baseline composition of the subjects’ gut microbiome could be used as a predictive tool for the short-term efficacy of metformin therapy and drug tolerance (35). Therefore, our study also analyzed the potential mechanism and pathways of the blood sugar-lowering effect of metformin from the perspectives of fecal metagenomics and metabolomics.

Several interesting observations were made. First, our data showed that the Simpson index was significantly higher in the probiotic group than in the placebo group after the intervention. In general, decreased microbiota diversity is considered an unhealthy state as it is indicative of a disrupted state of gut homeostasis, which frequently occurs due to external factors such as antibiotics and drug interference. Disturbances of the gut microbiota often affect the normal metabolism and immunity of the host. In clinical scenarios, reduced alpha diversity of gut microbes is commonly observed in various chronic diseases such as Parkinson’s disease and irritable bowel syndrome (36, 37). On the other hand, PCoA did not show a drastic shift in the gut microbiota structure, suggesting that the therapeutic effect of the administration of metformin with or without probiotics was not due to dramatic changes in the gut microbiota structure or composition.

To identify subtle but meaningful changes in the gut microbiome that are potentially related to symptom improvement, changes in responsive SGBs were analyzed. The second interesting observation was that the abundances of some beneficial SGBs, especially those related to SCFA production, were significantly increased in the probiotic group after the intervention, A previous study analyzed the fecal SCFA (including acetate, propionate, and butyrate) levels in 952 normoglycemic volunteers using two-way Mendelian randomization by combining genome-wide genotyping and intestinal metagenomic and metabolomic data, concluding that the increase in the gut production of SCFAs (especially butyrate) had great beneficial effects on the insulin response, energy balance, and metabolic homeostasis, but abnormalities in propionate metabolism could increase the risk of the development of diabetes, supporting that there is a causal effect of the gut microbiome on metabolic traits and the risk of T2DM (38). Another study performed in nonobese diabetic mice found that SCFAs could protect against autoimmune insulitis and slow the development of diabetes (39). The abundance and diversity of some acetate- and butyrate-producing bacteria are significantly related to the clinical effects of T2DM, and the increase in acetate production could promote the secretion of GLP-1 and peptide YY, thus improving blood glucose (40). Therefore, the increase in the abundance of SCFA-producing bacteria might improve host glucose metabolism, which is of particular importance in patients with diabetes.

Third, metabolic pathway analysis found that the differential metabolites between the probiotic group and the placebo group were related mainly to the pathways of tryptophan metabolism, primary BA biosynthesis, and sphingolipid metabolism. Consistently, after the 3-month intervention, the fecal contents of some BAs, chenodeoxycholic acid and hyodeoxycholic acid, in the probiotic group were significantly higher than those in the placebo group. Chenodeoxycholic acid and hyodeoxycholic acid are related compounds, as chenodeoxycholic acid can be converted to hyocholic acid by the action of the CYP3A4 enzyme. It is known that BAs can act as signaling molecules to regulate the metabolism of glucose, lipids, and energy (41). Moreover, Chaudhari et al. found that sleeve gastrectomy could increase a specific BA, cholic acid-7-sulfate, in the gastrointestinal tracts of obese patients and mice, acting as an agonist of G-protein-coupled bile acid receptor 1 (TGR5) to increase the GLP-1 level and upregulate TGR5 gene expression, thus increasing glucose tolerance and regulating blood sugar levels (42). Pig gallbladder has been used in traditional Chinese medicine to treat diabetes, and hyocholic acid is its active component for improving blood sugar homeostasis. Indeed, hyodeoxycholic acid is one of the components of hyocholic acids, and the synthetic precursors of both compounds are BAs produced by an alternative pathway. Hyocholic acid acts by activating TGR5 signaling, inhibiting farnesoid X receptor signaling, and upregulating the expression of proglucagon, thereby promoting GLP-1 production and secretion (43). Thus, our observation of elevated fecal levels of hyodeoxycholic acid and chenodeoxycholic acid after probiotic intervention could also be part of the mechanism of clinical symptom relief in the participants in this study.

**FIG 5:**
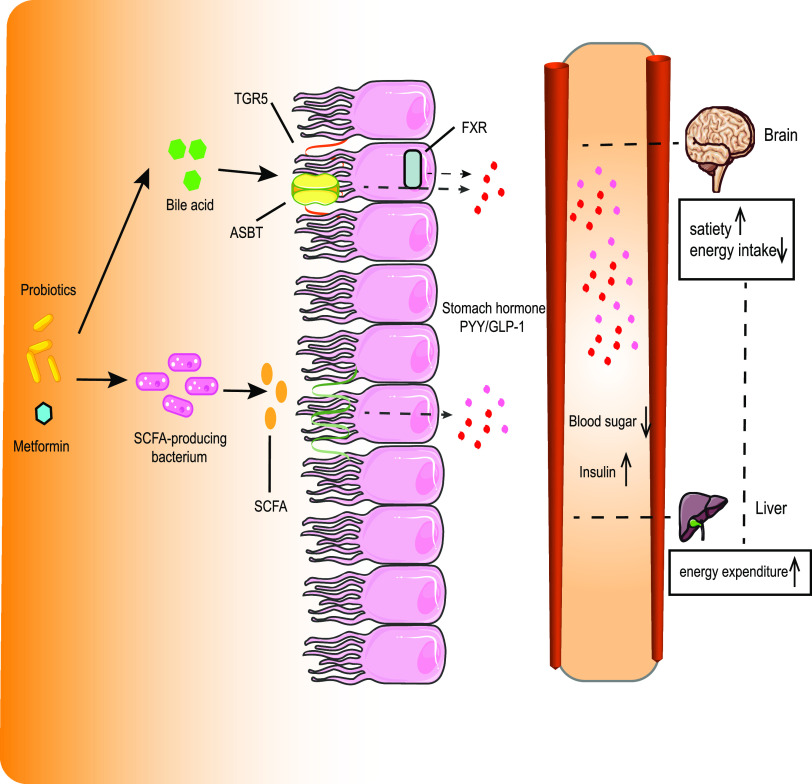
Schematic diagram showing the mechanism of clinical symptom improvement. Shown is a schematic representation of key pathways in the regulation of blood glucose metabolism and probiotic-driven host responses. SCFA, short-chain fatty acids; TGR5, G-protein-coupled bile acid receptor 1; FXR, farnesoid X receptor; ASBT, apical sodium-bile acid transporter; PYY, peptide YY; GLP-1, glucagon-like peptide 1.

Taken together, our results from metagenomics and metabolomics analyses supported that the coadministered probiotics acted synergistically with metformin to improve the clinical symptoms of T2DM patients possibly via two specific pathways (Fig. 5): (i) promoting SCFA-producing bacteria in the gut, thereby regulating the contents of colonic SCFAs and subsequently promoting GLP-1 secretion by L cells in the intestinal epithelium, and (ii) increasing the levels of specific BAs, thus activating G-protein-coupled receptors on the surface of L cells and nuclear receptors (such as farnesoid X receptor) and in turn promoting GLP-1 secretion. GLP-1 is a gastrointestinal hormone that also acts in the circulatory system together with metformin to reduce blood glucose levels. However, our proposed model needs to be verified further. Moreover, there was a notably differential loss of subjects from the start to the end of the trial, with more dropouts among those in the placebo arm, which could cause a bias in the results. Thus, the conclusions drawn in this study need to be interpreted carefully.

## MATERIALS AND METHODS

### Trial design.

A 3-month randomized double-blind RCT was performed. The clinical trial was performed in two phases, and a total of 58 patients who were clinically diagnosed with T2DM were recruited from the Inner Mongolia People’s Hospital (Hohhot, China) (Fig. 1A). Subjects were randomly assigned to the probiotic or the placebo group (1:1; *n* = 29 per group) based on randomization sequences generated by a computer-generated list. The randomization was performed by a statistician who had no contact with the participants. The subjects, doctors, and researchers involved in this study were kept unaware of these sequences until the completion of the study. After grouping, the probiotic group received Probio-X (3 × 10^10^ CFU/day, i.e., 2 g daily, taken after dinner) and metformin (0.75 g/day to 1.5 g/day, taken before three meals), while the placebo group received 2 g placebo powder (maltodextrin) (2 g daily, taken after dinner) and metformin (0.75 g/day to 1.5 g/day, taken before three meals). Probiotics and placebo materials were made in the form of dry powders, had the same appearance and taste, and were sealed in individually packed sachets (China Jinhua Yinhe Biotechnology Co., Ltd.) Probio-X contained five probiotic strains, namely, Lactobacillus casei Zhang, Bifidobacterium lactis V9, Lactobacillus plantarum P-8, Lactobacillus rhamnosus Probio-M9, and Bifidobacterium lactis Probio-M8.

### Ethics approval and consent to participate.

This study was approved by the Ethics Committee of the Health Department of the Inner Mongolia Autonomous Region and was registered at the China Clinical Trials Registry (http://www.chictr.org.cn/) (registration number ChiCTR2100050108). All volunteers signed informed consent before the trial.

### Subject recruitment.

Fifty-eight patients with T2DM were recruited. Patients were screened by inclusion and exclusion criteria. Inclusion criteria were that the patients (i) met the diagnostic criteria for T2DM in the Chinese guidelines for the prevention and treatment of T2DM (44), (ii) were aged 20 to 65 years, (iii) took metformin regularly or had taken other antidiabetic drugs but discontinued them for more than 3 months, (iv) had a duration of diabetes of ≥3 months, (v) had 6.5% ≤ HbA1c (hemoglobin A1c) ≤ 8.5%, and (vi) had taken no antibiotics within 1 month before the trial. Exclusion criteria were that the patients (i) had a history of major diseases, (ii) were pregnant or breastfeeding mothers, (iii) were hypertensive individuals whose blood pressure was still higher than 160/100 mm Hg even with regular treatment with two or more antihypertensive drugs, (iv) had a history of long-term use of glucocorticoids, (v) took probiotic products 1 month before the start of the trial, and (vi) failed to provide a complete sample of blood or stool. After screening for excluded patients and those who dropped out in the course of the trial, 48 people finally completed the RCT (probiotic group, *n* = 27; placebo group, *n* = 21). All patients were not informed of the products that they were taking throughout the trial, and antibiotics and other probiotic products were not taken during the RCT.

### Sample collection and clinical parameters.

All subjects were given a diabetes diet and activity guidance, understood the basic information about this RCT and their medical history, and provided information on their weight and waist circumference. All patients were instructed to visit the hospital every 4 weeks, and the patients’ body weight and waist circumference were recorded during each visit. Blood and fecal samples were taken at the start (month 0) and end (month 3) of the trial to monitor changes in relevant clinical parameters and the fecal microbiome and metabolome.

### Levels of clinical blood sugar indicators and insulin.

To determine the blood glucose levels of the patients, an HbA1c test, an oral glucose tolerance test, and an insulin release assay were performed. The HbA1c level was measured by a turbidimetric inhibition immunoassay. For the oral glucose tolerance test, standardized steamed bread from the hospital was used instead of a glucose solution as steamed bread is part of the regular Chinese diet and has been adopted for use in glucose tolerance testing in many Chinese hospitals. One major advantage of the use of a steamed bread meal is that it avoids nausea, vomiting, and other gastrointestinal discomforts caused by drinking a glucose solution, making it particularly suitable for patients who cannot tolerate oral glucose. For glucose tolerance tests and insulin release assays, the patients were fasting in the morning and did not take hypoglycemic drugs, 3 mL of fasting venous blood was drawn from the patients and centrifuged at 3,000 rpm for 10 min, and the supernatant was taken to detect fasting blood glucose and fasting insulin in time. Venous blood was drawn 0.5 h, 1 h, 2 h, and 3 h from the start of taking the first mouthful of steamed bread, and 3 mL of blood was drawn each time and centrifuged at 3,000 rpm for 10 min. The supernatant was collected, and blood glucose and insulin at each time point were measured over time. Blood glucose was detected by the hexokinase method (45), and insulin was detected by the electrochemiluminescence method (Cobas-E601; Roche Company). The remaining sera were stored in a −80°C refrigerator for later use.

Blood glucose and insulin concentrations were used to determine the homeostasis model assessment beta (HOMA-β) index, the homeostasis model assessment-estimated insulin resistance (HOMA-IR) index, the QUICKI, and the Gutt index (insulin sensitivity index [ISI_0,120_]) (31, 46–48) according to the equations HOMA-IR index = (FGB × FinS)/22.5, QUICKI = 1 / (lgI_0_ + lgG_0_), HOMA-β index = 20 × FinS/FPG − 3.5, and Gutt index (ISI_0,120_) = 75,000 + (G_0_ − G_120_) × 0.19 × BW/120 × G_mean(0,120)_ × log I_mean(0,120)_, where fasting plasma glucose (FPG) is expressed in millimoles per liter; fasting serum insulin (FinS) is expressed in microunits per milliliter; G_0_ and G_120_ are oral glucose tolerance test (OGTT) fasting and 120-min blood glucose levels (milligrams per deciliter), respectively; BW is body weight; and G_mean(0,120)_ and I_mean(0,120)_ are mean blood glucose (millimoles per liter) and mean insulin (microunits per milliliter) levels, respectively.

### Determination of levels of blood lipid, uric acid, and bile acids.

Dyslipidemia is common in people with diabetes, especially when blood sugar is poorly controlled. It manifests as increased TG, CHOL, and LDL-C levels and a decreased HDL-C level. Therefore, this study monitored these blood lipid indices. Venous blood (2 mL) taken from patients on an empty stomach for 8 to 12 h in the morning was collected and centrifuged at 3,000 × *g* for 10 min. The levels of CHOL, HDL-C, and LDL-C were measured by direct methods (49). Uric acid was detected by a peroxidase method. Bile acids play an important role in glucose metabolism. Therefore, LC-MS was used to determine the content of serum BAs (50).

### Metagenomic DNA extraction for shotgun metagenomic sequencing.

The QIAamp Fast DNA stool minikit (Qiagen GmbH, Hilden, Germany) was used to extract metagenomic DNA from fecal samples. The DNA concentration and quality were checked by using a Nanodrop spectrophotometer and the Qubit double-stranded DNA (dsDNA) assay kit in combination with a Qubit 2.0 fluorometer (Life Technologies, CA, USA), and 1% agarose gel electrophoresis was used to evaluate DNA integrity. Shotgun metagenomic sequencing was performed on all samples using an Illumina NovaSeq instrument, and the NEBNext Ultra DNA library prep kit (New England BioLabs [NEB]) was used to construct the library. All operations were done according to the manufacturers’ instructions. A total of 96 stool samples were subjected to shotgun sequencing (probiotics group, *n* = 27; placebo group, *n* = 21) (two time points for each subject). KneadData (v0.7.5) (http://huttenhower.sph.harvard.edu/kneaddata) was used to filter low-quality sequences.

### Read assembly, contig binning, genome dereplication, and taxonomic annotation.

The quality-controlled sequences were assembled into contigs using MEGAHIT (51). Contigs of >2,000 bp were selected for binning using the VAMB tool with default options. The completeness and contamination of metagenome-assembled genomes were evaluated using CheckM (https://github.com/Ecogenomics/CheckM). The high-quality genomes (completeness of ≥80% and contamination of ≤5%) were clustered, and the most representative genome from each replicate set was selected by dRep (52) for extracting species-level genome bins (SGBs) using the parameters -pa 0.95 and -sa 0.95. Finally, 304 SGBs were extracted from the pool of single representative genomes. The SGBs were annotated using the NCBI nonredundant nucleotide sequence database, and the predicted genes were searched against the UniProt Knowledgebase (UniProtKB) (release 2020.11) using the BLASTp function of DIAMON with default options. CheckM was used to calculate the relative abundance of each SGB, and the average content of SGBs in each contig was calculated and normalized as reads per kilobase per million (RPKM) by CoverM (https://github.com/wwood/CoverM). Next, sample diversity was calculated by using two R packages (vegan and optparse) based on the SGB abundance expressed in RPKM.

### Determination of fecal metabolites by LC-MS.

Stool samples (20 mg) were weighed and transferred to clean centrifuge tubes. A methanol-water internal standard extract (70%; 400 μL) was added, and the samples were mixed for 3 min, sonicated in an ice water bath for 10 min, further vortexed for 1 min, allowed to stand in a −20°C refrigerator for 30 min at 4°C, and centrifuged at 12,000 rpm for 10 min. The supernatant (300 μL) was transferred to a new centrifuge tube and centrifuged again at 12,000 rpm for 3 min. An aliquot of 200 μL of the supernatant was transferred to an injection bottle for analysis. Chromatographic and mass spectrometry conditions were set according to the conditions described in a previous study (53). During the instrumental analysis, a quality control (QC) sample (prepared by mixing an equal volume of each sample extract) was injected for LC-MS analysis after running each set of 15 samples to monitor the stability and reproducibility of the instrument and chromatographic conditions. The collected raw data were subjected to peak extraction, alignment, and retention time correction.

### Statistical analyses.

The graphic representation was generated using R software (v.4.0.2), Origin2019, and Adobe Illustrator. A Wilcoxon test and a *t* test were used to assess differences between groups for each variable, and a *P* value of <0.05 was considered statistically significant. For evaluating differences in the gut microbiota composition between groups, *P* values were corrected for multiple testing using the Benjamini-Hochberg procedure (54), and a corrected *P* value of <0.05 was considered statistically significant. ANOSIM, PCA, and PCoA (Bray-Curtis distance) were performed using R software (v.4.0.2). The quantitative data for the fecal metabolome were imported into MetaboAnalyst (https://www.metaboanalyst.ca/MetaboAnalyst) for multivariate statistical analysis, and differential metabolites were defined as those having a fold change of >2 and a *P* value of <0.05 or those having a fold change of <0.5 and a *P* value of <0.05. Information on the mass-to-charge ratios and retention times of the differential metabolites was used for identification using an in-house script, and the identified differential metabolites were searched using MetaboAnalyst to determine the respective differential metabolic pathways.

### Data availability.

The sequencing data generated in this study have been made available at the NCBI SRA under BioProject accession number PRJNA811727.
