# Supplementary material for: Adjunctive Probio-X Treatment Enhances the Therapeutic Effect of a Conventional Drug in Managing Type 2 Diabetes Mellitus by Promoting Short-Chain Fatty Acid-Producing Bacteria and Bile Acid Pathways
Source: mSystems. 2023 Jan 23;8(1):e01300-22. doi: 10.1128/msystems.01300-22 (PMC9948714; doi:10.1128/msystems.01300-22)
Supplement: TABLE S1 [file msystems.01300-22-s0002.pdf]

Table S1. Information of subjects

Subject number	Group	Subject Information						Years of illness	History of metformin use (≥ 3 months)	Metformin dose	Gastrointestinal effects
		Sample_ID	Gender	Age	Group	Weight (Kg)	Waistline (cm)				
1	Probiotic	Sample_A02	M	65	Probiotic	70	96	3 years	Yes	0.5 g, 3 times/day	NA
2	Probiotic	Sample_A03	M	42	Probiotic	90	93	1 year	Yes	0.5 g, 3 times/day	NA
3	Probiotic	Sample_A08	M	42	Probiotic	70	94	half a year	Yes	0.5 g, 3 times/day	NA
4	Probiotic	Sample_A10	M	38	Probiotic	82	92	More than 4 month	Yes	0.5 g, 3 times/day	NA
5	Probiotic	Sample_A12	F	31	Probiotic	76	89	1 year	Yes	0.5 g, 3 times/day	NA
6	Probiotic	Sample_A13	F	59	Probiotic	54	78	2 years	Yes	0.5 g, 3 times/day	NA
7	Probiotic	Sample_4A	M	52	Probiotic	94	102	3 years	Yes	0.5 g, 3 times/day	Stool regularly, once/morning
8	Probiotic	Sample_6A	M	49	Probiotic	82	102	More than 2 years	Yes	0.5 g, 3 times/day	No obvious change

9	Probiotic	Sample_7A	F	50	Probiotic	80	96	More than 20 years	Yes	0.5 g, 3 times/day	Improvement of constipation symptoms during the period of enrollment
10	Probiotic	Sample_9A	M	25	Probiotic	84	104	More than 4 month	Yes	0.5 g, 3 times/day	Improvement of constipation symptoms during the period of enrollment
11	Probiotic	Sample_10A	M	59	Probiotic	112	116	half a year	Yes	0.5 g, 3 times/day	NA
12	Probiotic	Sample_12A	F	64	Probiotic	80	104	12 years	Yes	0.5 g, 3 times/day	NA
13	Probiotic	Sample_13A	M	38	Probiotic	88	102	3 years	Yes	0.5 g, 3 times/day	NA
14	Probiotic	Sample_14A	M	62	Probiotic	82	102	1 year	Yes	NA	NA
15	Probiotic	Sample_16A	M	34	Probiotic	83	96	More than 4 months	Initial insulin therapy, now treated with metformin for more than three months	0.5 g, 3 times/day	Improvement of constipation symptoms during the period of enrollment

16	Probiotic	Sample_17A	F	59	Probiotic	62	78	8 years	Yes	0.5 g, 3 times/day	Improvement of constipation symptoms during the period of enrollment
17	Probiotic	Sample_19A	M	37	Probiotic	82	96	10 months	Yes	0.5 g, 3 times/day	No obvious change
18	Probiotic	Sample_20A	M	54	Probiotic	69	92	2 years	Yes	0.5 g, 3 times/day	NA
19	Probiotic	Sample_21A	M	60	Probiotic	111	117	More than half a year	Yes	0.5 g, 3 times/day	Improvement of constipation symptoms during the period of enrollment
20	Probiotic	Sample_31A	F	49	Probiotic	65	76	3 years	Yes	0.5 g, 3 times/day	Improvement of constipation symptoms during the period of enrollment
21	Probiotic	Sample_32A	F	51	Probiotic	67	86	More than 4 months	Yes	0.5 g, 3 times/day	Regularity of stool and improvement of stool character
22	Probiotic	Sample_35A	M	35	Probiotic	70	76	1 year	Yes	0.5 g, 3 times/day	No obvious change
23	Probiotic	Sample_36A	F	49	Probiotic	78	88	3 years	Yes	0.5 g, 3	Stool regularity

										times/day	
24	Probiotic	Sample_38A	M	59	Probiotic	72	82	More than half a year	Yes	0.5 g, 3 times/day	Improvement of stool character
25	Probiotic	Sample_39A	M	59	Probiotic	67	88	3 months	Yes	0.5 g, 3 times/day	Improvement of stool character
26	Probiotic	Sample_41A	M	53	Probiotic	78	85	6 years	Yes	0.5 g, 3 times/day	No obvious change
27	Probiotic	Sample_46A	F	40	Probiotic	85	92	More than half a year	Yes	0.5 g, 3 times/day	Stool regularity
28	Placebo	Sample_B01	M	61	placebo	87	102	5 years	Yes	0.5 g, 3 times/day	NA
29	Placebo	Sample_B08	F	56	placebo	70	88	3 years	Yes	0.5 g, 3 times/day	NA
30	Placebo	Sample_B10	M	47	placebo	78	93	3 years	Yes	0.5 g, 3 times/day	NA
31	Placebo	Sample_B12	M	42	placebo	101	100	2 years	Yes	0.5 g, 3 times/day	NA
32	Placebo	Sample_3B	F	44	placebo	82	92	half a year	Yes	0.5 g, 3 times/day	No obvious change
33	Placebo	Sample_5B	F	51	placebo	61	84	8 years	Yes	0.5 g, 3 times/day	No obvious change
34	Placebo	Sample_8B	F	53	placebo	57	92	3 years	Yes	0.25g, 3	No obvious

										times/day	change
35	Placebo	Sample_18B	M	36	placebo	77	82	One and a half years	Yes	0.5 g, 3 times/day	No obvious change
36	Placebo	Sample_22B	F	46	placebo	66	94	8 months	Yes	0.5 g, 3 times/day	No obvious change
37	Placebo	Sample_24B	F	63	placebo	65	87	Three and a half years	Yes	0.25 g, 3 times/day	Improvement of constipation symptoms during the period of enrollment
38	Placebo	Sample_26B	F	45	placebo	64	82	3 years	Yes	0.5 g, 3 times/day	Improvement of constipation symptoms during the period of enrollment
39	Placebo	Sample_28B	M	38	placebo	100	105	2 years	Yes	1.0 g/day	No obvious change
40	Placebo	Sample_29B	M	36	placebo	105	112	4 months	Yes	0.5 g, 3 times/day	No obvious change
41	Placebo	Sample_30B	M	26	placebo	83	89	1 year	Yes	0.5 g, 3 times/day	No obvious change
42	Placebo	Sample_33B	M	63	placebo	80	81	10 years	Yes	0.5 g, 3 times/day	No obvious change
43	Placebo	Sample_34B	M	32	placebo	71	85	4	Yes	0.5 g, 2	No obvious

								months		times/day	change
44	Placebo	Sample_37B	M	34	placebo	72	82	One and a half months	Yes	0.5 g, 2 times/day	No obvious change
45	Placebo	Sample_42B	F	59	placebo	63	74	One and a half years	Yes	0.5 g, 3 times/day	No obvious change
46	Placebo	Sample_43B	F	37	placebo	78	78	One and a half years	Insulin therapy during pregnancy, currently using metformin for more than three months	0.5 g, 2-3 times/day	Improvement of stool character
47	Placebo	Sample_44B	F	56	placebo	63	82	More than 2 years	Yes	0.5 g, 3 times/day	No obvious change
48	Placebo	Sample_45B	M	60	placebo	90	95	More than 9 months	Yes	0.5 g, 3 times/day	No obvious change
		P		0.60		0.44	0.16				
