# Supplementary material for: Adjunctive Probio-X Treatment Enhances the Therapeutic Effect of a Conventional Drug in Managing Type 2 Diabetes Mellitus by Promoting Short-Chain Fatty Acid-Producing Bacteria and Bile Acid Pathways
Source: mSystems. 2023 Jan 23;8(1):e01300-22. doi: 10.1128/msystems.01300-22 (PMC9948714; doi:10.1128/msystems.01300-22)
Supplement: TABLE S2 [file msystems.01300-22-s0003.pdf]

Table S2. Blood lipid indexes in diabetes patients before and after probiotics/placebo intervention

Lipid indicator	Amount of measured indicator (mean ± SD, mmol/L)				*P* value, Wilcoxon test	
	Probiotic, 0 month	Placebo, 0 month	Probiotic, 3 months	Placebo, 3 months	Probiotic, 0 month vs Probiotic, 3 months	Placebo, 0 month vs Placebo, 3 months
Triglyceride	2.31±1.75	2.25±1.17	2.16±0.94	1.95±1.09	0.61	0.38
High-density lipoprotein cholesterol	1.14±0.28	1.16±0.22	1.19±0.22	1.11±0.21	0.49	0.47
Low-density lipoprotein cholesterol	2.5±0.82	2.8±0.62	2.61±0.7	2.76±0.78	0.36	0.87
Total cholesterol	4.6±1.04	4.71±1.07	4.67±0.7	5.01±1.09	0.57	0.31
