# Supplementary material for: Adjunctive Probio-X Treatment Enhances the Therapeutic Effect of a Conventional Drug in Managing Type 2 Diabetes Mellitus by Promoting Short-Chain Fatty Acid-Producing Bacteria and Bile Acid Pathways
Source: mSystems. 2023 Jan 23;8(1):e01300-22. doi: 10.1128/msystems.01300-22 (PMC9948714; doi:10.1128/msystems.01300-22)
Supplement: TABLE S3 [file msystems.01300-22-s0004.pdf]

Table S3. Evaluation of islet function in diabetes patients before and after probiotic/placebo intervention

Glucose indicators	Amount of measured indicator (mean ± SD)				*P* value, Wilcoxon test	
	Probiotic, 0 month	Placebo, 0 month	Probiotic, 3 months	Placebo, 3 months	Probiotic, 0 month vs Probiotic, 3 months	Placebo, 0 month vs Placebo, 3 months
Area under the curve of blood sugar	31.26±7.33	31.68±8.33	31.17±8.16	31.35±9.38	0.73	0.95
Area under the curve of Insulin	105.31±40.21	125.96±80.33	135.36±62.76	115.23±60.62	0.15	0.86
Homeostasis model assessment-β (HOMA-β)	46.28±24.6	68.27±87.26	64.75±33.33	60.07±64.4	0.03	0.56
Homeostasis model assessment-estimated insulin resistance (HOMA-IR)	3.98±2.47	4.27±2.59	4.35±2.39	3.45±2.1	0.71	0.34
Quantitative insulin sensitivity check index (QUICKI)	0.54±0.08	0.53±0.06	0.53±0.07	0.55±0.06	0.71	0.34
Gutt index (insulin sensitivity index, ISI 0,120)	74989.47±9.24	74991.83±6.77	74988.16±7.84	74990.8±9.87	0.42	0.66
