# Supplementary material for: Adjunctive Probio-X Treatment Enhances the Therapeutic Effect of a Conventional Drug in Managing Type 2 Diabetes Mellitus by Promoting Short-Chain Fatty Acid-Producing Bacteria and Bile Acid Pathways
Source: mSystems. 2023 Jan 23;8(1):e01300-22. doi: 10.1128/msystems.01300-22 (PMC9948714; doi:10.1128/msystems.01300-22)
Supplement: TABLE S4 [file msystems.01300-22-s0005.pdf]

Table S4. Quality control information for metagenomic dataset

Group	Sampling time	Sample code	Raw_r1_reads	Raw_r1_bases	Raw_r2_reads	Raw_r2_bases	Final_r1_clean_reads	Final_r2_clean_bases	Final_r2_clean_reads	Final_r2_clean_bases	Final_clean_total_reads	Final_clean_total_bases
Probiotic	0 month	Sample_10A_1	19104093	2865613950	19104093	2865613950	18488877	2771615355	18488877	2771612851	36977754	5543228206
Probiotic	3 months	Sample_10A_2	21953076	3292961400	21953076	3292961400	21802909	3269634074	21802909	3269632302	43605818	6539266376
Probiotic	0 month	Sample_12A_1	28874562	4331184300	28874562	4331184300	28580706	4286804966	28580706	4286803624	57161412	8573608590
Probiotic	3 months	Sample_12A_2	21315326	3197298900	21315326	3197298900	21131185	3169253535	21131185	3169253487	42262370	6338507022
Probiotic	0 month	Sample_13A_1	22785203	3417780450	22785203	3417780450	22611602	3391533433	22611602	3391532025	45223204	6783065458
Probiotic	3 months	Sample_13A_2	24156531	3623479650	24156531	3623479650	23794898	3568963672	23794898	3568960286	47589796	7137923958
Probiotic	0 month	Sample_14A_1	23366694	3505004100	23366694	3505004100	23121878	3466538124	23121878	3466538319	46243756	6933076443
Probiotic	3 months	Sample_14A_2	21683143	3252471450	21683143	3252471450	21452367	3217343141	21452367	3217340530	42904734	6434683671
Probiotic	0 month	Sample_16A_1	23165037	3474755550	23165037	3474755550	22775476	3415538239	22775476	3415532810	45550952	6831071049
Probiotic	3 months	Sample_16A_2	22313556	3347033400	22313556	3347033400	21826790	3273352461	21826790	3273350347	43653580	6546702808
Probiotic	0 month	Sample_17A_1	29661059	4449158850	29661059	4449158850	28939807	4339909196	28939807	4339906620	57879614	8679815816

Probiotic	3 months	Sample_17A_2	28369028	4255354200	28369028	4255354200	26932693	4038005022	26932693	4037998631	53865386	8076003653
Placebo	0 month	Sample_18B_1	29085785	4362867750	29085785	4362867750	28611309	4289907971	28611309	4289901327	57222618	8579809298
Placebo	3 months	Sample_18B_2	21853869	3278080350	21853869	3278080350	21640160	3245131850	21640160	3245130125	43280320	6490261975
Probiotic	0 month	Sample_19A_1	22077027	3311554050	22077027	3311554050	21681100	3251607678	21681100	3251602555	43362200	6503210233
Probiotic	3 months	Sample_19A_2	29807122	4471068300	29807122	4471068300	29139246	4370223378	29139246	4370214547	58278492	8740437925
Probiotic	0 month	Sample_21A_1	25614921	3842238150	25614921	3842238150	25337729	3799328303	25337729	3799339944	50675458	7598668247
Probiotic	3 months	Sample_20A_2	22709709	3406456350	22709709	3406456350	22319565	3347318814	22319565	3347313229	44639130	6694632043
Probiotic	0 month	Sample_20A_1	22443449	3366517350	22443449	3366517350	21998969	3298236068	21998969	3298236887	43997938	6596472955
Probiotic	3 months	Sample_21A_2	25435200	3815280000	25435200	3815280000	24707098	3705444709	24707098	3705440842	49414196	7410885551
Placebo	0 month	Sample_22B_1	24180726	3627108900	24180726	3627108900	23536389	3528237825	23536389	3528235605	47072778	7056473430
Placebo	3 months	Sample_22B_2	23259325	3488898750	23259325	3488898750	21028757	3153574364	21028757	3153577499	42057514	6307151863
Placebo	0 month	Sample_24B_2	17662940	2649441000	17662940	2649441000	17461116	2618360527	17461116	2618359581	34922232	5236720108
Placebo	3 months	Sample_24B_1	25782557	3867383550	25782557	3867383550	25006056	3749315846	25006056	3749311285	50012112	7498627131

Placebo	0 month	Sample_26B_1	18760483	2814072450	18760483	2814072450	18509495	2775813872	18509495	2775812577	37018990	5551626449
Placebo	3 months	Sample_26B_2	21124715	3168707250	21124715	3168707250	20827504	3123679838	20827504	3123678935	41655008	6247358773
Placebo	0 month	Sample_28B_1	23915588	3587338200	23915588	3587338200	23426511	3512789889	23426511	3512783472	46853022	7025573361
Placebo	3 months	Sample_28B_2	26418694	3962804100	26418694	3962804100	26176413	3925938064	26176413	3925937222	52352826	7851875286
Placebo	0 month	Sample_29B_1	20274385	3041157750	20274385	3041157750	18614081	2791237106	18614081	2791231374	37228162	5582468480
Placebo	3 months	Sample_29B_2	22799049	3419857350	22799049	3419857350	22562164	3383758500	22562164	3383754798	45124328	6767513298
Probiotic	0 month	Sample_31A_1	22633432	3395014800	22633432	3395014800	22445851	3366485811	22445851	3366485884	44891702	6732971695
Placebo	3 months	Sample_30B_1	22858456	3428768400	22858456	3428768400	22620595	3392469260	22620595	3392463643	45241190	6784932903
Placebo	0 month	Sample_30B_2	25408809	3811321350	25408809	3811321350	25171499	3775143999	25171499	3775146461	50342998	7550290460
Probiotic	3 months	Sample_31A_2	24315201	3647280150	24315201	3647280150	24099642	3614629209	24099642	3614634344	48199284	7229263553
Probiotic	0 month	Sample_32A_1	24928073	3739210950	24928073	3739210950	24592400	3688422350	24592400	3688423392	49184800	7376845742
Probiotic	3 months	Sample_32A_2	28659796	4298969400	28659796	4298969400	28201784	4228266437	28201784	4228271591	56403568	8456538028
Placebo	0 month	Sample_33B_1	23196584	3479487600	23196584	3479487600	23012910	3450442347	23012910	3450443542	46025820	6900885889

Placebo	3 months	Sample_33B_2	22391145	3358671750	22391145	3358671750	21986885	3297535564	21986885	3297527225	43973770	6595062789
Placebo	0 month	Sample_34B_1	22243292	3336493800	22243292	3336493800	21854337	3277542355	21854337	3277540588	43708674	6555082943
Placebo	3 months	Sample_34B_2	20156383	3023457450	20156383	3023457450	19830405	2974237362	19830405	2974233167	39660810	5948470529
Probiotic	0 month	Sample_35A_1	20932308	3139846200	20932308	3139846200	20652013	3097348417	20652013	3097342523	41304026	6194690940
Probiotic	3 months	Sample_35A_2	23311893	3496783950	23311893	3496783950	22986834	3447696176	22986834	3447690938	45973668	6895387114
Probiotic	0 month	Sample_36A_1	21665820	3249873000	21665820	3249873000	21484722	3222443654	21484722	3222441582	42969444	6444885236
Probiotic	3 months	Sample_36A_2	22468451	3370267650	22468451	3370267650	22242854	3335397575	22242854	3335404326	44485708	6670801901
Placebo	0 month	Sample_37B_1	31323769	4698565350	31323769	4698565350	30727491	4608322461	30727491	4608314826	61454982	9216637287
Placebo	3 months	Sample_37B_2	18586918	2788037700	18586918	2788037700	18452817	2767656534	18452817	2767656038	36905634	5535312572
Probiotic	0 month	Sample_38A_1	20712289	3106843350	20712289	3106843350	20536583	3078398410	20536583	3078399140	41073166	6156797550
Probiotic	3 months	Sample_38A_2	23663741	3549561150	23663741	3549561150	23330650	3498944231	23330650	3498947091	46661300	6997891322
Probiotic	0 month	Sample_39A_1	22804542	3420681300	22804542	3420681300	22573260	3385509332	22573260	3385505402	45146520	6771014734
Probiotic	3 months	Sample_39A_2	23310981	3496647150	23310981	3496647150	23039800	3455728858	23039800	3455725151	46079600	6911454009

Placebo	0 month	Sample_3B_1	25587318	3838097700	25587318	3838097700	23460536	3518627586	23460536	3518622815	46921072	7037250401
Placebo	3 months	Sample_3B_2	22193170	3328975500	22193170	3328975500	21919859	3286693151	21919859	3286693287	43839718	6573386438
Placebo	0 month	Sample_42B_1	22201764	3330264600	22201764	3330264600	21982021	3296658026	21982021	3296656058	43964042	6593314084
Probiotic	3 months	Sample_41A_2	21420277	3213041550	21420277	3213041550	21091821	3163368244	21091821	3163366389	42183642	6326734633
Probiotic	0 month	Sample_41A_1	21771130	3265669500	21771130	3265669500	20837186	3124911691	20837186	3124909630	41674372	6249821321
Placebo	3 months	Sample_42B_2	20927701	3139155150	20927701	3139155150	20746826	3111311413	20746826	3111312626	41493652	6222624039
Placebo	0 month	Sample_43B_1	21288011	3193201650	21288011	3193201650	21106054	3165592180	21106054	3165589467	42212108	6331181647
Placebo	3 months	Sample_43B_2	20722215	3108332250	20722215	3108332250	20560646	3083465222	20560646	3083461838	41121292	6166927060
Placebo	0 month	Sample_44B_1	21110673	3166600950	21110673	3166600950	20914511	3136627554	20914511	3136630050	41829022	6273257604
Placebo	3 months	Sample_44B_2	22036788	3305518200	22036788	3305518200	21743061	3260110245	21743061	3260110702	43486122	6520220947
Placebo	0 month	Sample_45B_1	22158673	3323800950	22158673	3323800950	21958661	3290543586	21958661	3290543188	43917322	6581086774
Placebo	3 months	Sample_45B_2	21279244	3191886600	21279244	3191886600	21099097	3162567626	21099097	3162566571	42198194	6325134197
Probiotic	0 month	Sample_46A_1	22975206	3446280900	22975206	3446280900	22728692	3407213374	22728692	3407214150	45457384	6814427524

Probiotic	3 months	Sample_46A_2	22423560	3363534000	22423560	3363534000	22222263	3333001970	22222263	3333002533	44444526	6666004503
Probiotic	0 month	Sample_4A_1	24366336	3654950400	24366336	3654950400	24102646	3614971717	24102646	3614969488	48205292	7229941205
Probiotic	3 months	Sample_4A_2	21257180	3188577000	21257180	3188577000	21052768	3157683531	21052768	3157681559	42105536	6315365090
Probiotic	0 month	Sample_6A_1	24187866	3628179900	24187866	3628179900	23983963	3596939621	23983963	3596938845	47967926	7193878466
Placebo	3 months	Sample_5B_1	24000257	3600038550	24000257	3600038550	23744795	3561465564	23744795	3561463320	47489590	7122928884
Placebo	0 month	Sample_5B_2	22916098	3437414700	22916098	3437414700	22656025	3397147702	22656025	3397149047	45312050	6794296749
Probiotic	3 months	Sample_6A_2	22208668	3331300200	22208668	3331300200	21968382	3294926460	21968382	3294920148	43936764	6589846608
Probiotic	0 month	Sample_7A_1	22169860	3325479000	22169860	3325479000	21998393	3298451699	21998393	3298449224	43996786	6596900923
Probiotic	3 months	Sample_7A_2	22188983	3328347450	22188983	3328347450	21948095	3289768435	21948095	3289770282	43896190	6579538717
Placebo	0 month	Sample_8B_1	20770666	3115599900	20770666	3115599900	20287131	3040246201	20287131	3040250411	40574262	6080496612
Placebo	3 months	Sample_8B_2	25548489	3832273350	25548489	3832273350	25187597	3772660321	25187597	3772657881	50375194	7545318202
Probiotic	0 month	Sample_9A_1	24315660	3647349000	24315660	3647349000	24013806	3601533856	24013806	3601535370	48027612	7203069226
Probiotic	3 months	Sample_9A_2	24575867	3686380050	24575867	3686380050	24258132	3638167011	24258132	3638165093	48516264	7276332104

Probiotic	0 month	A02-1	23763573	3534807797	23763573	3536294248	23338711	3490760102	23338711	3490517254	46677422	6981277356
Probiotic	3 months	A02-2	25434417	3775783603	25434417	3773227192	24861724	3715243448	24861724	3713467619	49723448	7428711067
Probiotic	0 month	A03-1	23735747	3535924662	23735747	3534556409	23373607	3497914030	23373607	3496144982	46747214	6994059012
Probiotic	3 months	A03-2	16374818	2435709777	16374818	2435112888	16070993	2403930428	16070993	2402878323	32141986	4806808751
Probiotic	0 month	A08-1	24319248	3612874452	24319248	3613088458	23857888	3565479029	23857888	3563201166	47715776	7128680195
Probiotic	3 months	A08-2	28384405	4219877366	28384405	4210821075	27831242	4160977943	27831242	4154160624	55662484	8315138567
Probiotic	0 month	A10-1	20851023	3104800684	20851023	3100673341	20418827	3055264823	20418827	3052990071	40837654	6108254894
Probiotic	3 months	A10-2	20774457	3096314842	20774457	3093961244	20467895	3063812428	20467895	3061897511	40935790	6125709939
Probiotic	0 month	A12-1	30587357	4545838836	30587357	4538951841	29986471	4482393191	29986471	4476370551	59972942	8958763742
Probiotic	3 months	A12-2	27626534	4105924430	27626534	4094415805	27054412	4043993009	27054412	4036181708	54108824	8080174717
Probiotic	0 month	A13-1	22969830	3417720096	22969830	3420359710	22593420	3379791911	22593420	3379791516	45186840	6759583427
Probiotic	3 months	A13-2	26398078	3925461846	26398078	3906848173	25831586	3862193859	25831586	3851356119	51663172	7713549978
Placebo	0 month	B01-1	28024574	4162285883	28024574	4159399219	27458341	4103312763	27458341	4099364810	54916682	8202677573

Placebo	3 months	B01-2	29341439	4360874791	29341439	4353486294	28765828	4300093967	28765828	4293685312	57531656	8593779279
Placebo	0 month	B08-1	25941296	3866642977	25941296	3861421056	25527843	3821343515	25527843	3818128941	51055686	7639472456
Placebo	3 months	B08-2	23661755	3529430639	23661755	3514761188	23270349	3484293997	23270349	3477709113	46540698	6962003110
Placebo	0 month	B10-1	23272880	3468041588	23272880	3462983777	22871123	3423086654	22871123	3419806475	45742246	6842893129
Placebo	3 months	B10-2	27858491	4140075294	27858491	4138123930	27336055	4086103893	27336055	4082341518	54672110	8168445411
Placebo	0 month	B12-1	23614987	3520539422	23614987	3513628423	23251068	3480645508	23251068	3476949038	46502136	6957594546
Placebo	3 months	B12-2	25370795	3784527870	25370795	3750662607	24827163	3717045916	24827163	3702985011	49654326	7420030927
