# Supplementary material for: Adjunctive Probio-X Treatment Enhances the Therapeutic Effect of a Conventional Drug in Managing Type 2 Diabetes Mellitus by Promoting Short-Chain Fatty Acid-Producing Bacteria and Bile Acid Pathways
Source: mSystems. 2023 Jan 23;8(1):e01300-22. doi: 10.1128/msystems.01300-22 (PMC9948714; doi:10.1128/msystems.01300-22)
Supplement: TABLE S5 [file msystems.01300-22-s0006.pdf]

Table S5. Analysis of similarities (ANOSIM) to evaluate differences in gut microbiota structure

Group1	Group2	P	R
Probiotic, 0 month	Probiotic, 3 months	0.761	0.0174
Probiotic, 0 month	Placebo, 0 month	0.103	0.0424
Placebo, 0 month	Placebo, 3 months	0.989	0.0434
Probiotic, 3 months	Placebo, 3 months	0.076	0.048
