# Supplementary material for: Adjunctive Probio-X Treatment Enhances the Therapeutic Effect of a Conventional Drug in Managing Type 2 Diabetes Mellitus by Promoting Short-Chain Fatty Acid-Producing Bacteria and Bile Acid Pathways
Source: mSystems. 2023 Jan 23;8(1):e01300-22. doi: 10.1128/msystems.01300-22 (PMC9948714; doi:10.1128/msystems.01300-22)
Supplement: TABLE S6 [file msystems.01300-22-s0007.pdf]

Table S6. Significantly differential species-level genome bins (SGBs)

SGBs ID	Taxonomy	Relative abundance (mean ± SD, %)				*P* value, Wilcoxon test			
		Probiotic, 0 month	Probiotic, 3 months	Placebo, 0 month	Placebo, 3 months	Probiotic, 0 month vs Placebo, 0 month	Probiotic, 0 month vs Probiotic, 3 months	Probiotic, 3 months vs Placebo, 3 months	Placebo, 0 month vs Placebo, 3 months
Sample_39A_2.bin_3	*Fusicatenibacter saccharivorans*	5.64±7.05	6.35±8.59	7.43±11.44	1.59±1.97	0.74	0.80	0.03	0.01
Sample_35A_2.bin_6	uncultured *Butyricicoccus* sp.	1.46±1.55	1.31±1.5	1.01±1.15	0.6±0.9	0.36	0.72	0.04	0.18
Sample_13A_1.bin_4	*Eubacterium* sp. CAG:274	2.08±4.33	4.28±7.22	1.93±6.88	0.86±2.15	0.23	0.24	0.04	0.81
Sample_42B_1.bin_7	*Ruminococcus bromii*	2.79±6.37	3.77±7.54	1.79±3.35	0.44±1.1	0.91	0.42	0.04	0.25
Sample_14A-2.bin_1	*Faecalibacterium prausnitzii*	2.12±3.7	5.64±7.27	2.88±3.17	2±3.29	0.23	0.05	0.10	0.36
Sample_A13-1.bin_1	*Bifidobacterium longum*	4.92±9.59	5.16±11.03	1.26±2.43	1.01±2.88	0.11	0.87	0.03	0.50
