# Supplementary figures and images for: Adjunctive Probio-X Treatment Enhances the Therapeutic Effect of a Conventional Drug in Managing Type 2 Diabetes Mellitus by Promoting Short-Chain Fatty Acid-Producing Bacteria and Bile Acid Pathways
Source: mSystems. 2023 Jan 23;8(1):e01300-22. doi: 10.1128/msystems.01300-22 (PMC9948714; doi:10.1128/msystems.01300-22)

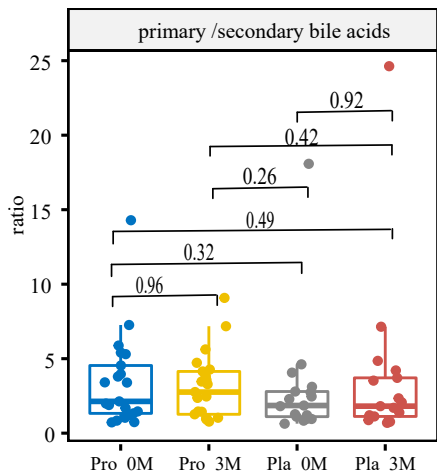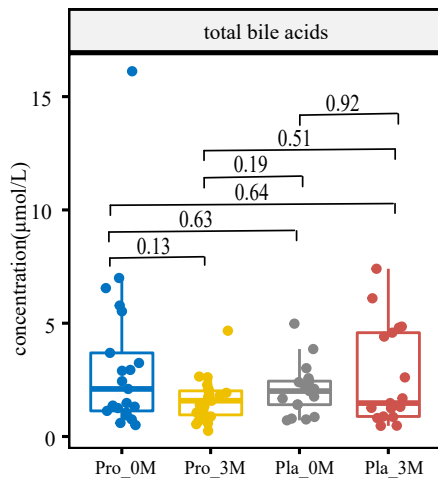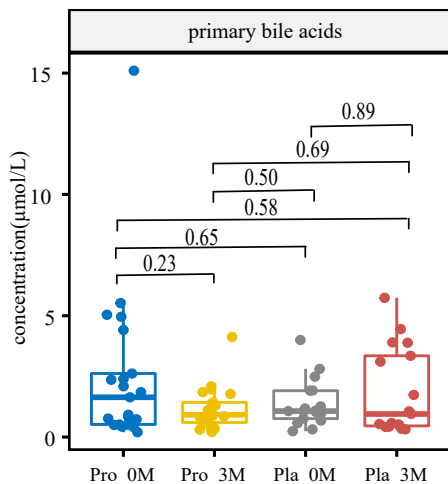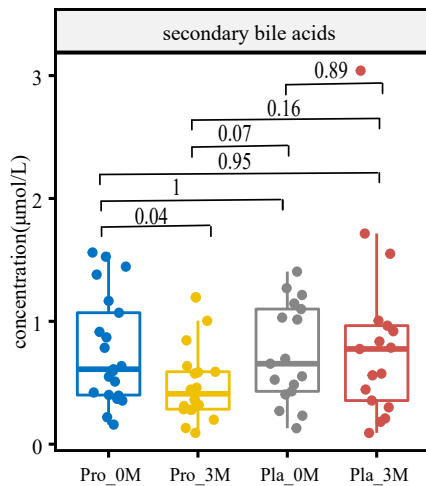

Supplement: FIG S1 [file msystems.01300-22-s0001.pdf]
