# Supplementary material for: Adjunctive Probio-X Treatment Enhances the Therapeutic Effect of a Conventional Drug in Managing Type 2 Diabetes Mellitus by Promoting Short-Chain Fatty Acid-Producing Bacteria and Bile Acid Pathways
Source: mSystems. 2023 Jan 23;8(1):e01300-22. doi: 10.1128/msystems.01300-22 (PMC9948714; doi:10.1128/msystems.01300-22)
Supplement: TABLE S7 [file msystems.01300-22-s0008.pdf]

Table S7. Differential fecal metabolites identified by liquid chromatography-mass spectrometry

Metabolite ID	Mean signal intensity (arbitrary unit)				*P* value, Wilcoxon test			
	Probiotic, 0 month	Probiotic, 3 months	Placebo, 0 month	Placebo, 3 months	Probiotic, 0 month vs Placebo, 0 month	Probiotic, 0 month vs Probiotic, 3 months	Probiotic, 3 months vs Placebo, 3 months	Placebo, 0 month vs Placebo, 3 months
Sucralose	31617.83	27494.96	68175.99	61338.53	0.10	0.46	0.03	0.97
Chenodeoxycholic acid	94311.81	124641.83	95020.94	61629.45	0.58	0.09	0.01	0.63
Cholic acid	263151.95	184172.06	94550.74	57284.95	0.18	0.74	0.05	0.59
Cortisol	113099.21	77983.79	64547.82	32542.47	0.84	0.68	0.02	0.15
Sphingosine	298129.99	294642.77	192792.32	64397.89	0.89	0.78	0.05	0.02
5-hydroxytryptophan	134104.92	146660.04	112907.61	52005.81	0.84	0.56	0.02	0.10
Hyodeoxycholic acid	41153.62	54232.02	43271.05	19633.18	0.64	0.34	0.00	0.01
D-tryptophan	114446.47	95909.87	60353.45	40038.89	0.34	0.56	0.01	0.45
